# Valorization of Fermented Shrimp Waste with Supercritical CO_2_ Conditions: Extraction of Astaxanthin and Effect of Simulated Gastrointestinal Digestion on Its Antioxidant Capacity

**DOI:** 10.3390/molecules26154465

**Published:** 2021-07-24

**Authors:** Luis Angel Cabanillas-Bojórquez, Erick Paul Gutiérrez-Grijalva, Gustavo Adolfo González-Aguilar, Leticia Xochitl López-Martinez, Ramón Ignacio Castillo-López, Pedro de Jesús Bastidas-Bastidas, José Basilio Heredia

**Affiliations:** 1 Centro de Investigación en Alimentación y Desarrollo, A. C. Carretera a Eldorado Km 5.5 Col. Campo El Diez, Culiacán CP 80110, Sinaloa, Mexico; luis.cabanillasdc18@estudiantes.ciad.mx (L.A.C.-B.); pbastidas@ciad.mx (P.d.J.B.-B.); 2 Cátedras CONACyT-Centro de Investigación en Alimentación y Desarrollo, A. C. Carretera a Eldorado Km 5.5 Col. Campo El Diez, Culiacán CP 80110, Sinaloa, Mexico; erick.gutierrez@ciad.mx; 3 Centro de Investigación en Alimentación y Desarrollo, A. C. CTAOV, Carretera Gustavo Enrique Astiazarán Rosas, No. 46, Col. La Victoria, Hermosillo CP 83304, Sonora, Mexico; gustavo@ciad.mx; 4 Cátedras CONACyT-Centro de Investigación en Alimentación y Desarrollo, A. C. Carretera Gustavo Enrique Astiazarán Rosas, No. 46, Col. La Victoria, Hermosillo CP 83304, Sonora, Mexico; leticia.lopez@ciad.mx; 5 Facultad de Ciencias Químico Biológicas, Universidad Autónoma de Sinaloa, Ciudad Universitaria, Culiacán CP 80013, Sinaloa, Mexico; ricastil@uas.edu.mx

**Keywords:** astaxanthin, shrimp waste, supercritical CO_2_ extraction, antioxidant activity, bioaccessibility, optimization

## Abstract

Lactic acid fermentation increases the bioactive properties of shrimp waste. Astaxanthin is the principal carotenoid present in shrimp waste, which can be found esterified in the liquid fraction (liquor) after its lactic acid fermentation. Supercritical CO_2_ technology has been proposed as a green alternative to obtain astaxanthin from fermented shrimp waste. This study aimed to optimize astaxanthin extraction by supercritical CO_2_ technology from fermented liquor of shrimp waste and study bioaccessibility using simulated gastrointestinal digestion (GD) of the optimized extract. A Box–Behnken design with three variables (pressure, temperature, and flow rate) was used to optimize the supercritical CO_2_ extraction. The optimized CO_2_ extract was obtained at 300 bar, 60 °C, and 6 mL/min, and the estimated characteristics showed a predictive extraction yield of 11.17%, antioxidant capacity of 1.965 mmol of Trolox equivalent (TE)/g, and astaxanthin concentration of 0.6353 µg/g. The experiment with optimal conditions performed to validate the predicted values showed an extraction yield of 12.62%, an antioxidant capacity of 1.784 mmol TE/g, and an astaxanthin concentration of 0.52 µg/g. The astaxanthin concentration decreased, and the antioxidant capacity of the optimized extract increased during gastrointestinal digestion. In conclusion, our optimized supercritical CO_2_ process is suitable for obtaining astaxanthin from shrimp by-products after lactic acid fermentation.

## 1. Introduction

Shellfish are a widely and increasingly consumed food, and shrimp are economically the most important [1]. However, shrimp processing produces a large amount of waste, since approximately 60% of their weight is considered waste. Shrimp waste mainly consists of the head, tail, and cephalothorax [2,3,4,5] and is a good source of protein, ash, chitin, lipids, and astaxanthin, which are considered important industrial compounds [6,7]. Astaxanthin is the principal carotenoid present in shrimp waste (head and cephalothorax). It is an important scavenging free radical and has reportedly 10-fold more antioxidant activity than other carotenoids such as β-carotene and 500 times higher antioxidant activity than tocopherol (vitamin E) [8,9,10].

Astaxanthin also has neuroprotective, anti-inflammatory, anti-diabetic, and cardioprotective properties, attenuates photo-toxicity caused by UV radiation, and has recently shown anticancer activity [4,11,12,13,14]. In addition, astaxanthin is found in nature in three forms: free, esterified, or a complex with proteins (carotenoprotein) and lipoproteins (carotenolipoproteins), the free form being the most unstable [5,12,15]. In crustaceans, astaxanthin is found in the carotenoprotein form, which has different properties than other forms [5]. Like other carotenoids, astaxanthin can form aggregates with polar solvents, which have medical applications since they depend on interaction with other molecules [16].

In general, to exert their bioactive properties, antioxidants must be bioaccessible and/or bioavailable. Bioaccessibility refers to the amount of a compound present in the gut after being released from the food matrix. Complementarily, bioavailability is associated with compounds that have been absorbed, distributed, metabolized, and excreted from the body. To date, not many studies have investigated the bioaccessibility and bioavailability of astaxanthin. The available information states that both free and esterified forms of astaxanthin have low bioaccessibility and bioavailability due to their susceptibility to thermal and chemical degradation [17,18]. Yang, Gu, Luan, Qiao, Cao, Xue and Xu [17] showed that pure astaxanthin ester in oil matrixes increases the overall stability of the molecule and its antioxidant activity.

It has also been reported that the bioaccessibility and bioavailability of astaxanthin can be modified due to its conformational isomers because *cis*- and *trans*-astaxanthin can permeate lipid membranes to a different extent both in vitro and in vivo. However, conformational isomers of esterified astaxanthin have different bioaccessibility, since they are able to cross the epithelial barrier and stabilize the molecule; therefore, esterified astaxanthin could be more bioaccessible and have different health benefits [5,12,16].

Several methods are used for astaxanthin extraction from shrimp waste, such as lactic acid fermentation [19], solvent extraction [20], enzymatic hydrolysis [21], and supercritical CO_2_ extraction [15]. Lactic acid fermentation is a technologically flexible, economical, and eco-friendly method that can separate shrimp components such as astaxanthin [7,22], which is in the liquid fraction, also called liquor [23]. However, liquor is rich in other compounds such as lipids, proteins, and ash; therefore, it is necessary to use some efficient extraction technique to separate astaxanthin from them [22,23]. Supercritical CO_2_ extraction has shown high astaxanthin extraction yield and recovery; this technique has some advantages compared to other extraction methods, such as eco-friendliness, faster extraction, high purity, and thermolabile molecule recovery [5,24].

Therefore, this study aimed to optimize the supercritical CO_2_ extraction of astaxanthin from lyophilized liquor obtained by lactic acid fermentation of shrimp waste, analyze its antioxidant activity, and determine its bioaccessibility level using an in vitro model.

## 2. Results

### 2.1. Predictive Models

Three predictive models were obtained to fit the quadratic polynomial of Equation (2) to experimental data of the effects of different supercritical CO_2_ extraction variables on the three response variables (extraction yield, antioxidant capacity, and astaxanthin concentration), as shown in Table 1. These predictive models were fit by analysis of variance (ANOVA; Table 1). The extraction yield varied from 6.895% to 13.421%, the antioxidant capacity varied from 0.3565 to 1.5247 mmol/g lyophilized liquor, and the astaxanthin concentration varied from 0.2538 to 0.6311 µg/g lyophilized liquor.

#### 2.1.1. Extraction Yield

According to ANOVA (Supplementary data, Table S1), the extraction yield model was highly significant for its small *p*-value (<0.0001), in addition to the lack of fit being insignificant (*p* > 0.05). The model was dependent on the linear term of pressure (*P*; *p* < 0.05), temperature (*T*; *p* < 0.01), and flow rate (*F*; *p* < 0.05), as well as the interaction between temperature and flow rate (*T* × *F*; *p* < 0.01). Predictive models for extraction yield (*Y_Y_*) are shown in Equations (1) and (2).

Using coded variables:

(1) $$Y_{Y}=9.49+0.42X_{1}-0.73X_{2}-0.43X_{3}+0.43\;X_{2}X_{3}$$

Using original variables:

(2) $$Y_{Y}=36.99+5.6398\times 10^{-3}P-0.5583\;T-6.2817\;F+0.1213\;T\;F$$

**Figure 1:**
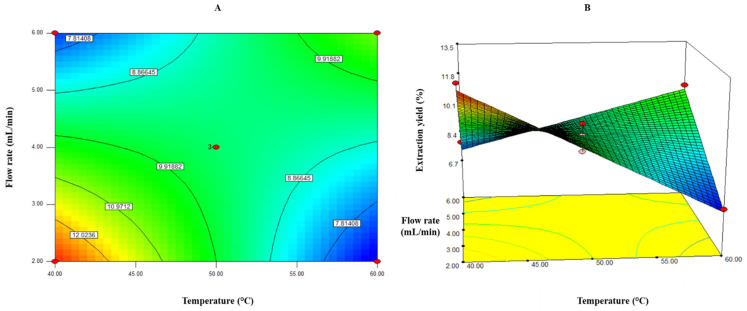
Contour plots (**A**) and response surface (**B**) for the effect of temperature (°C) and flow rate (mL/min) on the extraction yield of supercritical CO_2_ extraction of lyophilized liquor.

This predictive model explained 90.14% of the total variation (*p* < 0.05) in extraction yield values (Table 2). In addition, the variation coefficient between experimental data and the results predicted by the model was 6.11%. Milán–Carrillo, Montoya–Rodríguez, Gutiérrez–Dorado, Perales–Sánchez and Reyes–Moreno [25] reported that a good predictive model should meet the following criteria: adjusted R^2^ (coefficient of determination) ≥ 0.8, a significant level *p* < 0.05, coefficient of variance (CV) ≤ 0.1, and lack-of-fit test > 0.1. These values indicate that the model is adequate and reproducible [26]. The maximum extraction yield values were observed at a temperature of 40–45 °C and a flow rate of 2–3 mL/min (Figure 1).

#### 2.1.2. Antioxidant Capacity

The antioxidant capacity (ABTS assay) model was highly significant for its small *p*-value (<0.0001), and the lack of fit was insignificant (*p* > 0.05) (Supplementary data, Table S2). The model was dependent on the linear term of pressure (*P*; *p* < 0.05), temperature (*T*; *p* < 0.0001), and flow rate (*F*; *p* < 0.001), as well as the interaction between pressure and flow rate (*P* × *F*; *p* < 0.0001) and temperature and flow rate (*T* × *F*; *p* < 0.0002). Predictive models for antioxidant capacity (*Y_AC_*) are shown in Equations (3) and (4).

Using coded variables:

(3) $$Y_{AC}=0.89-0.033X_{1}+0.21X_{2}+0.15X_{3}++0.40X_{1}X_{3}+0.24X_{2}X_{3}$$

Using original variables:

(4) $$Y_{AC}=4.41-0.011P-0.026T-1.11F+2.663\times 10^{-3}P\;T+0.011\;T\;F$$

**Figure 2:**
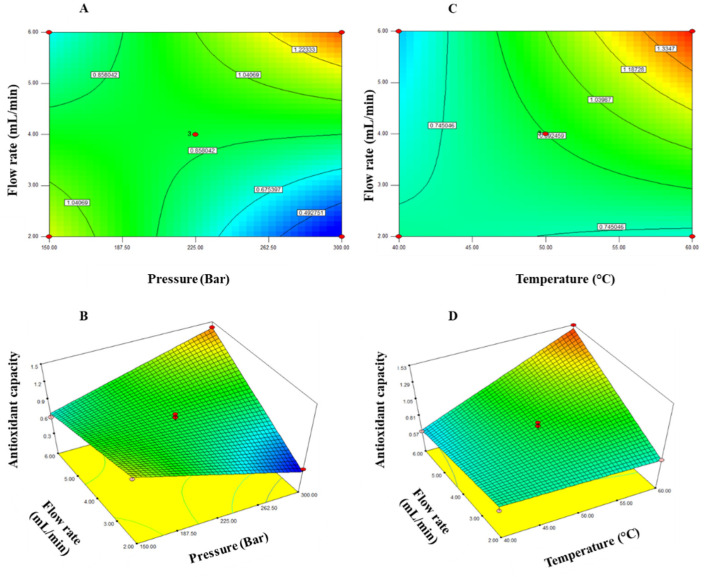
Contour plots (**A**) and response surface (**B**) for the effect of pressure (bar) and flow rate (mL/min) as well as contour plots (**C**) and response surface (**D**) for the effect of temperature (°C) and flow rate (mL/min) on the antioxidant capacity (ABTS assay) of supercritical CO_2_ extraction of lyophilized liquor.

This predictive model explained 93.42% of the total variation (*p* < 0.05) in antioxidant capacity values (Table 2). In addition, the variation coefficient between experimental data and the results predicted by the model was 8.22%. These values indicated that the model was adequate and reproducible. The maximum values of antioxidant capacity were observed at a pressure of 262.5–300 bar and a temperature of 55–60 °C, at a pressure of 150–187.5 bar and a flow rate of 2–3 mL/min, and at a temperature of 55–60 °C and a flow rate of 5–6 mL/min (Figure 2).

#### 2.1.3. Astaxanthin Concentration

The analysis of variance for astaxanthin concentration (Supplementary data, Table S3) demonstrated that the model was significantly (*p* < 0.0001) dependent on linear terms of pressure (*P*; *p* < 0.0001), temperature (*T*; *p* < 0.05), and flow rate (*F*; *p* < 0.0001), as well as the interaction between pressure and flow rate (*P* × *F*; *p* < 0.0004). Predictive models for astaxanthin concentration (*Y_AST_*) are shown in Equations (5) and (6).

Using coded variables:

(5) $$Y_{AST}=0.40+0.073X_{1}-8.904\times 10^{-3}X_{2}+0.90X_{3}+0.080X_{1}X_{3}$$

Using original variables:

(6) $$Y_{AST}=0.5231-1.1489\times 10^{-3}P-8.9039\times 10^{-4}T-0.0743F+5.3131\times 10^{-4}P\;F$$

**Figure 3:**
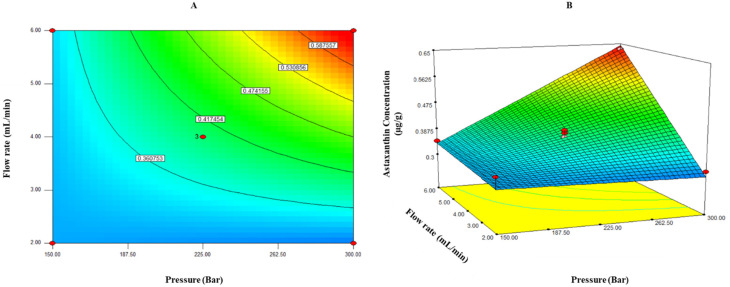
Contour plots (**A**) and response surface (**B**) for the effect of pressure (bar) and flow rate (mL/min) on the astaxanthin concentration of supercritical CO_2_ extraction of lyophilized liquor.

This predictive model explained 93.34% of the total variation (*p* < 0.05) in astaxanthin concentration values (Table 2). Furthermore, the relative dispersion of the experimental points from the models’ predictions (CV) was found to be 7.72%. These values indicated that the model is adequate and reproducible. The maximum values of astaxanthin concentration were observed at a pressure of 262.5–300 bar and a flow rate of 5–6 mL/min (Figure 3).

### 2.2. Optimization

**Figure 4:**
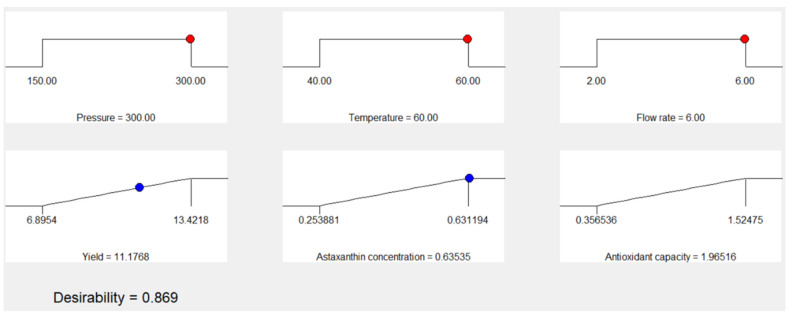
Response optimization of the effect of pressure, temperature, and flow rate for CO_2_ supercritical extraction on extraction yield, antioxidant capacity (ABTS assay), and astaxanthin concentration of lyophilized liquor.

In this study, numerical optimization found the optimal levels of pressure, temperature, and flow rate for CO_2_ supercritical extraction for determining the highest extraction yield, antioxidant capacity, and astaxanthin concentration of lyophilized liquor. The optimal CO_2_ supercritical extraction of lyophilized liquor was at a pressure of 300 bar, a temperature of 60 °C, and a flow rate of 6 mL/min (Figure 4). The global desirability was 0.869; according to previous studies, the optimum value is 1, and a global desirability value of >0.6 is considered acceptable [25,27]. Under optimal conditions, the predictive response values were an extraction yield of 11.17%, an antioxidant capacity of 1.965 mmol TW/g, and an astaxanthin concentration of 0.6353 µg/g (Table 2). The experiment with optimal conditions to validate the predictive models showed an extraction yield of 12.62% ± 0.1%, an antioxidant capacity of 1.784 ± 0.081 mmol TE/g, and an astaxanthin concentration of 0.52 ± 0.04 µg/g.

### 2.3. Antioxidant Characterization of Optimized Supercritical CO_2_ Extraction 

The antioxidant capacity of the optimized CO_2_ extract obtained from lyophilized liquor was evaluated using two assays (ABTS and ORAC) (Table 3). The antioxidant capacities of the optimized CO_2_ extract evaluated by ABTS assay were 1.784 ± 0.08 mmol TE/g lyophilized liquor, while the antioxidant capacity evaluated by ORAC assay was 5.44 ± 0.47 mmol TE/g lyophilized liquor. Furthermore, after the gastrointestinal digestion, the highest antioxidant capacity was found after intestinal digestion (13.73 mmol TE/g lyophilized liquor) and gastric digestion (59.09 ± 3.01 mmol TE/g lyophilized liquor) by the ABTS and ORAC assays, respectively; the lowest antioxidant capacity was in the undigested extract.

### 2.4. Bioaccessibility of Astaxanthin of Optimized CO_2_ Extract

As shown in Table 3, the highest astaxanthin concentration was observed in the undigested sample with 0.52 ± 0.04 µg/g lyophilized liquor. The simulated gastrointestinal digestion had a detrimental effect on the astaxanthin content of the sample with a loss of around 63% of its original content in the gastric digesta. Moreover, astaxanthin was not found in the intestinal digesta. 

## 3. Discussion

Supercritical CO_2_ extraction is a separation technology based on the solubility of bioactive compounds in a fluid whose density and dissolving power can be modified by different parameters such as pressure, temperature, and flow rate [10]. During this process, the bioactive compounds are solubilized and diffused from the matrix to the solvent by diffusive and convective transport processes [15,28]. Supercritical CO_2_ extraction has some advantages compared with other separation methods, such as high bioactive compound purity, enhanced extraction efficiency, and low environmental impact [10,15]. For example, astaxanthin has been extracted from shrimp waste using supercritical CO_2_ extraction [2,10,15,29,30]. In addition, liquor obtained from lactic acid fermentation is a source of lipophilic compounds, such as astaxanthin, which is separated from the carotenoprotein complex by proteolytic enzymes generated by lactic acid bacteria during the process [7,23,31]. Therefore, astaxanthin from fermented liquor of shrimp waste can be extracted using supercritical CO_2_ extraction. The process is affected by pressure, temperature, and flow rate, consistent with previous reports on the extraction of lipophilic compounds from shrimp waste by supercritical CO_2_ extraction [15,30]. In addition, the extraction yield and antioxidant capacity of the supercritical extracts are affected by different conditions due to the extraction of lipophilic compounds such as astaxanthin and lipids by the modified density of supercritical CO_2_ fluid and co-solvent addition (ethanol) [4,15].

The optimized supercritical CO_2_ extract obtained here showed a higher extraction yield than those reported by Sánchez–Camargo, Martinez–Correa, Paviani and Cabral [30], Razi Parjikolaei et al. [32], and Roy, Getachew, Cho, Park, and Chun [4] and a similar extraction yield as that of Yang et al. [33]. In addition, the optimized supercritical CO_2_ extract showed similar antioxidant capacity, by ABTS and ORAC assays, like that reported by Roy, Getachew, Cho, Park, and Chun [4], which has been related to lipophilic compounds such as carotenoids (mostly astaxanthin) [4,10,15]. However, the optimized supercritical CO_2_ extract from fermented liquor of shrimp waste showed a lower astaxanthin concentration comparison with the supercritical CO_2_ extract from shrimp waste without lactic acid fermentation, as reported by Radzali, Masturah, Baharin, Rashidi, and Rahman [29], Sánchez–Camargo, Martinez–Correa, Paviani and Cabral [30], Razi Parjikolaei, Casas Cardoso, Fernández Ponce, Mantell, Fretté, and Christensen [32], and Radzali et al. [34]. The lower concentration of astaxanthin obtained in the present study could be associated with the different factors that affect the astaxanthin concentration in shrimp. For instance, the food matrix can affect extraction due to the different carotenoid–protein interactions that affect astaxanthin release. In addition, the carotenoid concentration varies depending on the shrimp species, age, and production area. Thus, studies using supercritical CO_2_ extraction can have different astaxanthin yields [4,15,35].

The bioaccessibility of carotenoids such as astaxanthin is often limited by their interaction with food matrix constituents. For instance, astaxanthin in shrimp waste is usually found in a chemical complex with proteins linked by the -OH group of the xanthophyll molecule. The bioaccessibility of astaxanthin of marine origin has been evaluated in salmon and supplements by Chitchumroonchokchai and Failla [36]. Contrary to our results, astaxanthin in raw salmon flesh and supplements is relatively stable during in vitro digestion. Nonetheless, the bioaccessibility of carotenoids is also associated with their origin and chemical characteristics [37]. Many studies have focused on the increased stability of astaxanthin using different matrix formulations to protect the molecule in the gastrointestinal environment [37,38,39]. During lactic acid fermentation of shrimp waste, astaxanthin from fermented liquor is found in the free or esterified form [15,16,40]. Thus, it seems that lactic acid fermentation produces more bioaccessible astaxanthin; however, the astaxanthin in our study was released from its carotenoprotein matrix and thus is more susceptible to chemical degradation due to pH changes during gastrointestinal digestion [16,17,41].

The simulated gastrointestinal digestion significantly affected (*p* < 0.05) the astaxanthin concentration and antioxidant capacity (ABTS and ORAC assays). Several studies have shown that the initial concentration of bioactive compounds can be degraded due to the physicochemical and biochemical factors in the gastrointestinal environment. In in vitro studies, these detrimental effects can be attributed mainly to pH and enzymatic conditions. Due to pH changes, bioactive compounds such as carotenoids can be partially hydrolyzed and deprotonated, affecting their reactivity toward the probes used in antioxidant studies [42]. This can be the reason the antioxidant capacity of optimized supercritical CO_2_ extracts increased at the end of gastrointestinal digestion. In addition, metabolic changes during gastrointestinal digestion can lead to the formation of new metabolites or deprotonated forms of the original bioactive molecule. This is yet to be elucidated, and astaxanthin metabolites during GD have not yet been reported [43,44].

The astaxanthin concentration decreases during gastrointestinal digestion, while its antioxidant capacity increases. This can be related to the fact that astaxanthin can be found in marine products as different isomers. The distribution and proportion of astaxanthin isomers are related to their bioaccessibility, bioavailability, and bioactivity [45]. For instance, both *trans*- and *cis*-astaxanthin isomers are found in shrimp waste [16]. Further studies are needed in order to evaluate the isomer profile of astaxanthin in fermented liquor obtained by optimized supercritical CO_2_ extraction. Therefore, the decrease in the astaxanthin concentration in the optimized extract during gastrointestinal digestion could be due to the predominant *cis* isomer since it has a lower capacity to permeate the lipid membrane than the *trans* isomer [16,46]. Further studies are needed to prove this hypothesis.

Supercritical CO_2_ extraction is efficient in extracting lipophilic compounds (such as lipids) and carotenoids (such as β-carotene, α-carotene, β-cryptoxanthin, esterified astaxanthin, and free astaxanthin) from shrimp waste, which all have adequate and, in conjunction, good antioxidant capacity [2,15]. Therefore, the increase in the antioxidant capacity during gastrointestinal digestion of the optimized supercritical CO_2_ extract could be due to the available carotenoids released with good antioxidant activity [2]. Moreover, encapsulation strategies using different encapsulation matrixes have been developed to increase the bioaccessibility and bioavailability of astaxanthin of marine origin [17,37,38,39]. Therefore, the low bioaccessibility of astaxanthin in our study supports the need to continue these encapsulation studies to increase the bioaccessibility of this antioxidant carotenoid of interest in the pharmaceutical, food, and food supplement industries.

## 4. Materials and Methods

### 4.1. Biological Material

Shrimp (*Litopenaeus vannamei*) exoskeleton was purchased from a local market in Culiacán, Sinaloa, Mexico. The exoskeleton was washed with tap water and ground. Then, a commercial mixture of lactic acid bacteria (*Streptococcus thermophilus*, *Lactobacillus bulgaricus*, *Lactococcus lactis*, *Bifidobacterium infantis*, *Lactobacillus acidophilus*, and *Leuconostoc mesenteroides*) needed during cheese production (Bioprox, Ika-lac, CDMX, Mexico) and molasses production (obtained from a sugarcane-processing facility from Sinaloa, Mexico) were used for lactic acid fermentation of the shrimp waste, according to Marcia, Malespín, Sánchez and Benavente [47], with slight modifications. Briefly, 400 g of ground shrimp waste was mixed with molasses and whey in a 1:5 ratio (obtained by the separation of cheese from a commercial mixture of lactic acid bacteria previously inoculated with commercial milk), obtaining a final volume of 2 L in a batch reactor (BioFlo 120; Eppendorf, Hamburg, Germany) at 20 °C without agitation under anaerobic conditions, 8.4 °Brix, and a fermentation time of 108 h. This modified method is under a patenting process (RGP-DDAJ-27446). After fermentation, the liquor (liquid fraction of the fermentation product) was lyophilized at −50 °C and 0.070 mPa for 7 days in a freeze dryer (LABCONCO FreeZone 18; Labconco Corporation, MO, USA), and the lyophilized solid was stored under darkness at −20 °C until further use.

### 4.2. Reagents and Chemicals

Analytical-grade ethanol, methanol, hydrochloric acid, sodium carbonate, phosphoric acid, potassium phosphate monobasic, potassium phosphate dibasic, and potassium persulfate were purchased from Sigma-Aldrich (St. Louis, MO, USA). Astaxanthin standard (Sigma SML0982, with purity ≥97 for HPLC isolated from *Blakeslea trispora*), 2,2′-azino-bis-(3-ethylbenzothiazoline-6-sulphonic acid) (ABTS) (Sigma 10102946001), 2,2′-azobis(2-methylpropionamidine) dihydrochloride (AAPH) (Sigma 440914), 6-hydroxy-2,5,7,8-tetramethylchroman-2-carboxylic acid (Trolox) (Sigma 238813), and fluorescein (Sigma 46955) were also purchased from Sigma-Aldrich (St. Louis, MO, USA).

### 4.3. Supercritical CO_2_ Extraction

Astaxanthin extraction was performed according to Roy, Getachew, Cho, Park, and Chun [4], with modifications, using an MV-10 ASFE extractor (Waters Corporation, Milford, MA, USA), with CO_2_ as a solvent and ethanol (99.9%) as a co-solvent in a 95:5 ratio. According to the experimental design (Table 1), the conditions used were chosen based on the literature for astaxanthin extraction from shrimp waste and preliminary studies (not shown). A 1.5 g sample of lyophilized liquor was placed in a 10 mL vessel; extraction was carried out at different pressures, temperatures, and flow rates (Table 4). Every extraction was carried out at a dynamic extraction time of 2 h. After each extraction, the extract was centrifugated using a HERMLE centrifuge (HERMLE Z 36 HK; Labortechnik, Wehingen, Baden-Wurtemberg, Germany) at 9390× *g* (rotor 221.22) and at 4 °C for 15 min. The collected supernatant was separated and dried using a rotary evaporator (BÜCHI Labortechnik R-215; BÜCHI Labortechnik AG, Flawil, Switzerland), nitrogen (gas) was used for complete samples drying in darkness to prevent the extract from oxidation. All samples were resuspended in 1 mL of ethanol for antioxidant capacity assays; other extracts were resuspended in 1 mL ethyl acetate for astaxanthin concentration and stored in darkness at −20 °C for further analysis.

### 4.4. Extraction Yield

The following equation was used to determine the extraction yield:

(7) $$Extraction\;yield\;\left(\%\right)=\frac{m\;\left(extract\right)}{m\;\left(initial\right)}$$

where *m* (*extract*) is the dry mass obtained from supercritical CO_2_ extraction and *m* (*initial*) is the weight of lyophilized liquor used for the extraction [4]. 

### 4.5. Radical-Scavenging Activity

#### 4.5.1. ABTS

The 2,2-azino-di(3-ethylbenzthiazoline-6-sulfonate) (ABTS) radical-scavenging activity was evaluated according to Karadag, Ozcelik, and Saner [48] with slight modifications. Briefly, 10 µL of the extract resuspended with ethanol was mixed with 190 µL of ABTS solution (7.4 mM ABTS, 2.6 mM K_2_S_2_O_8_, and ethanol) and incubated for 2 h at room temperature in darkness. The absorbance at 734 nm was measured using an Epoch microplate spectrophotometer (Bio-Tek Instruments, Inc., Winooski, VT, USA), and ethanol was used as a blank. The results were expressed as Trolox equivalent millimoles per gram (mmol TE/g) of lyophilized liquor.

#### 4.5.2. ORAC

The antioxidant capacity was evaluated using the oxygen radical absorbance capacity (ORAC) assay according to Huang, Ou, Hampsch-Woodill, Flanagan, and Prior [49] with slight modifications. Briefly, 25 µL of supercritical CO_2_ extract resuspended in ethanol was mixed with 200 µL of fluorescein and 75 µL of 2,2′-azobis(2-methylpropionamidine) dihydrochloride (AAPH). The absorbance of elicitation and emission was set at 485 and 580 nm, respectively. The reaction absorbance was monitored for 70 min using a Synergy HT spectrometer (Bio-Tek Instruments, Inc., Winooski, VT, USA), and a phosphatic solution (mixture of phosphoric acid, potassium phosphate monobasic, and potassium phosphate dibasic) was used as a blank. The results were expressed as Trolox equivalent millimoles per gram (mmol TE/g) of lyophilized liquor.

### 4.6. Astaxanthin Content

The astaxanthin concentration in supercritical CO_2_ extracts of lyophilized liquor was analyzed according to Hu, Lu, Lv, Wang, Ding, and Wang [50] with slight modifications. Supercritical extracts resuspended in ethyl acetate were filtered using a membrane filter (0.45 µm) and injected in a Varian HPLC system (Palo Alto, CA, USA) including a Varian 9012 solvent delivery instrument, a Varian 9050 variable wavelength UV–VIS detector, and a Rheodyne 7161 manual injector of a 20 µL loop sample. In addition, Star chromatography workstation version 6.0 was used to analyze data. A chromatographic Phenomenex Kinetex C18 column (250 mm × 4.6 mm, 5 µm) (Phenomenex, Torrance, CA, USA) was used for compound separation. The flow rate, detection wavelength, temperature, and injection volume were 0.8 mL/min, 474 nm, 25 °C, and 20 µL, respectively. The mobile phase was a solution of acetonitrile/methanol/dichloromethane in a 80:15:5 (*v*/*v*/*v*) ratio. Astaxanthin identification was performed by comparing the retention time of the sample with the astaxanthin standard. A standard curve of astaxanthin (R2 = 0.992) was obtained for the injection of six concentrations (0.22, 0.55, 1.10, 4.97, 9.95, and 22.11 µg/mL). The astaxanthin concentration in supercritical CO_2_ extracts was calculated using the integrated area of HPLC peak areas and expressed as micrograms of astaxanthin per gram (µg/g) of lyophilized liquor.

### 4.7. Experimental Design 

The Box–Behnken design with a three-variable set of 15 experiments (Table 1) was used to optimize the supercritical CO_2_ extraction. Pressure (X1), temperature (X2), and flow rate (X3) were considered independent variables, and extraction yield, antioxidant capacity (ABTS), and astaxanthin concentration were considered dependent variables. Individual experiments were performed in random order. A quadratic polynomial regression model was assumed to fit (*Y*) the obtained data. The following model form was developed to describe the three response surfaces (*Y*):

(8) $$Y=\beta_{0}+\overset{3}{\underset{i=0}{\sum }}\beta_{i}X_{i}+\overset{3}{\underset{i=0}{\sum }}\beta_{ii}X_{i}^{2}+\sum \overset{3}{\underset{i<j=0}{\sum }}\beta_{ij}X_{i}X_{j}$$

where *Y* is the predicted response variable value (extraction yield, antioxidant capacity, or astaxanthin concentration), *β*_0_ is the constant value, *β*_1_ and *β*_2_ are linear coefficients, *β*_12_ is the interaction coefficient, and β_11_ and *β*_22_ are quadratic coefficients. The significant terms (*p* ≤ 0.05) for the second-order polynomial model were recalculated to obtain a predictive model for each variable [25]. All the results were analyzed using statistical software (Design Expert version 7.0.0; Stat-Ease Inc., Minneapolis, MN, USA) to determine the optimum conditions for supercritical CO_2_ extraction. The optimal levels were obtained by the numerical method and by contour plots of the graphs. The supercritical CO_2_ extraction at optimum conditions was carried out for quintupled. 

### 4.8. Simulated Gastrointestinal Digestion 

The optimized CO_2_ extract resuspended in ethanol was subjected to in vitro simulated gastrointestinal digestion according to the static method described by Brodkorb, Egger, Alminger, Alvito, Assunção, Ballance, Bohn, Bourlieu-Lacanal, Boutrou, Carrière, Clemente, Corredig, Dupont, Dufour, Edwards, Golding, Karakaya, Kirkhus, Le Feunteun, Lesmes, Macierzanka, Mackie, Martins, Marze, McClements, Ménard, Minekus, Portmann, Santos, Souchon, Singh, Vegarud, Wickham, Weitschies, and Recio [51] with slight modifications. This procedure simulated digestion in the mouth, stomach, and small intestine using a model of the human digestive tract that mimics the chemical composition and pH of digestive fluids at 37 °C. In the final process, the samples were centrifugated at 9390× *g* at 4 °C for 15 min, and the supernatant was collected and freeze-dried. For further analysis, the digestive extracts were resuspended in ethanol (for antioxidant capacity analysis) and ethyl acetate (for astaxanthin concentration study).

### 4.9. Calculation of Bioaccessibility 

Bioaccessibility was calculated using the formula

(9) $$Bioaccessibility\;\left(\%\right)=\frac{Initial\;value}{Final\;value}\times 100$$

The initial values are the initial antioxidant activity and astaxanthin concentration of the undigested samples. The final value is the antioxidant activity and astaxanthin concentration after in vitro gastrointestinal digestion. 

### 4.10. Statistical Analysis

Antioxidant capacity (ABTS and ORAC), extraction yield, and astaxanthin concentration data of optimized CO_2_ extracts were reported as the mean ± standard deviation. Digestive extract data were analyzed by one-way analysis of variance (ANOVA), followed by Turkey’s HSD test using the statistical package Minitab 17 (Minitab Inc., State College, PA, USA). Statistical differences at the level of *p* < 0.05 were considered significant. Each experiment was performed in quintuple or as otherwise specified. 

## 5. Conclusions

This study optimized the supercritical CO_2_ extraction of astaxanthin from lactic-acid-fermented liquor from shrimp waste. The optimized extracts showed antioxidant activity against different free radicals, indicating their possible use as additives during the development of functional foods or food supplements. However, the simulated gastrointestinal digestion process decreased the astaxanthin concentration, mainly attributed to pH changes during each digestive stage. Although the astaxanthin concentration decreased, its content did not reflect the antioxidant capacity of the optimized extracts since they showed higher antioxidant potential. This may also suggest that optimized supercritical CO_2_ extracts are rich in other carotenoids that can act in synergy and could be available during gastrointestinal digestion. Furthermore, encapsulation strategies could be implemented to improve the bioaccessibility of astaxanthin and its derivatives. Finally, supercritical CO_2_, considered a green technology for the extraction of bioactive compounds, is recommended for future studies on the valorization of astaxanthin from waste biomass of marine origin. 

## Figures and Tables

**Table 1:** Regression coefficients and variance analysis of second-order polynomial models for supercritical CO_2_ extraction of lyophilized liquor.

Regression Coefficients	Extraction Yield (%)		Antioxidant Capacity (ABTS) (mmol TE/g Lyophilized Liquor)		Astaxanthin Concentration (µg/g Lyophilized Liquor)	
	Coded	Uncoded	Coded	Uncoded	Coded	Uncoded
Intercept						
β_0_	9.49	36.99	0.89	4.41	0.40	0.5231
Linear						
β_1_	0.42	5.6398 × 10^−3^	−0.033	−0.011	0.073	−1.1489 × 10^−3^
β_2_	−0.73	−0.5583	0.21	−0.026	−8.903 × 10^−3^	−8.9039 × 10^−4^
β_3_	−0.43	−6.2817	0.15	−1.11	0.090	−0.0743
Interaction						
β_12_						
β_13_			0.40	2.66 × 10^−3^	0.080	5.3131 × 10^−4^
β_23_	0.43	0.1213	0.24	0.011		
Statistical parameters						
R^2^	0.9014	0.9014	0.9342	0.9342	0.9334	0.9334
Regression (*p* value)	0.0001	0.0001	0.0001	0.0001	0.0001	0.0001
Lack of fit (*p* value)	0.8241	0.8241	0.0686	0.0686	0.1073	0.1073

**Table 2:** Confirmation report of CO_2_ supercritical extraction of lyophilized liquor.

Factor	Name	Optimum Level	Low Level	High Level	Coding
A	Pressure (bar)	300	150	300	Actual
B	Temperature (°C)	60	40	60	Actual
C	Flow rate (mL/min)	6	2	6	Actual
**Response**	**Predicted Mean**	**Measured Data Mean**	**SE Prediction**	**95% PI Low**	**95% PI High**
Extraction yield (%)	11.17	12.62	0.75	9.5	12.86
Antioxidant capacity (ABTS) (mmol TE/g)	1.965	1.784	0.11	1.71	2.22
Astaxanthin concentration (µg/g)	0.6353	0.52	0.04	0.55	0.73

**Table 3:** Astaxanthin concentration by HPLC and antioxidant capacity (ABTS and ORAC assays) of optimized undigested and digested supercritical CO_2_ extracts of lyophilized liquor.

Digestion Phase	Astaxanthin Concentration (µg/g Lyophilized Liquor)	Bioaccessibility of Astaxanthin (%)	ABTS (mmol TE/g Lyophilized Liquor)	Changes in ABTS Values during GD (%)	ORAC (mmol TE/g Lyophilized Liquor)	Changes in ORAC Values during GD (%)
Undigested	0.52 ± 0.04 ^a^	-	1.78 ± 0.08 ^d^	-	5.44 ± 0.47 ^d^	-
Oral	0.48 ± 0.04 ^a^	92.30% ^a^	6.59 ± 0.32 ^b^	370.22% ^b^	21.14 ± 2.07 ^c^	388.6% ^c^
Gastric	0.19 ± 0.01 ^b^	36.53% ^b^	3.99 ± 0.28 ^c^	224.15% ^c^	59.09 ± 3.01 ^a^	1,086.21% ^a^
Intestinal	ND		13.73 ± 0.83 ^a^	771.34% ^a^	49.60 ± 2.09 ^b^	911.76% ^b^

^a,b,c,d^ Means that do not share a letter are significantly different according to Tukey’s test (*p* < 0.05). ND: not detected; TE: Trolox equivalent.

**Table 4:** Box–Behnken design of factors with codes for astaxanthin supercritical CO_2_ extraction from lyophilized liquor.

Sample Number	Pressure(bar)	Temperature (°C)	Flow Rate (mL/min)	Extraction Yield (%)	Antioxidant Capacity (ABTS) (mmol ET/g Lyophilized Liquor)	Astaxanthin Concentration (µg/g Lyophilized Liquor)
1	150	40	4.00	9.309	0.8834	0.3374
2	300	40	4.00	10.015	0.5051	0.5024
3	150	60	4.00	8.317	0.9964	0.2787
4	300	60	4.00	8.510	1.0859	0.4482
5	150	50	2.00	9.841	1.145	0.3534
6	300	50	2.00	10.616	0.3565	0.3197
7	150	50	6.00	8.414	0.5996	0.3460
8	300	50	6.00	10.123	1.4093	0.6311
9	225	40	2.00	13.421	0.7061	0.2538
10	225	60	2.00	6.895	0.7132	0.3270
11	225	40	6.00	7.806	0.5743	0.5158
12	225	60	6.00	10.986	1.5247	0.4843
13	225	50	4.00	9.421	0.9421	0.3928
14	225	50	4.00	8.553	0.9366	0.4068
15	225	50	4.00	10.088	0.9934	0.4157

## Data Availability

Not applicable.

## Supplementary Materials

The following are available online. Table S1. ANOVA analysis for extraction yield. Table S2. ANOVA analysis for antioxidant capacity. Table S3. ANOVA analysis for astaxanthin concentration.
