# Supplementary material for: Valorization of Fermented Shrimp Waste with Supercritical CO2 Conditions: Extraction of Astaxanthin and Effect of Simulated Gastrointestinal Digestion on Its Antioxidant Capacity
Source: Molecules. 2021 Jul 24;26(15):4465. doi: 10.3390/molecules26154465 (PMC8348114; doi:10.3390/molecules26154465)
Supplement: Supplementary file 1 [file molecules-26-04465-s001.zip › molecules-1261448-supplementary.pdf]

Supplementary data

Table S1. ANOVA analysis for extraction yield

Source		Sum of Squares	df (Degrees of freedom)	Mean Square	F Value	p-value Prob > F	
Model		30.7367	4	7.6842	22.8536	<0.0001	Significant
A-Pressure		1.4313	1	1.4313	4.2570	0.066	
B-Temperature		4.2692	1	4.2692	12.6970	0.0052	
C-Flow rate		1.4828	1	1.4828	4.4099	0.0621	
BC		23.5535	1	23.5535	70.0506	<0.0001	
Residual		3.3624	10	0.3362			
	Lack of Fit	2.1775	8	0.2722	0.4594	0.8241	Not significant
	Pure error	1.1848	2	0.5924			
Cor Total		34.0991	14				

Table S2. ANOVA analysis for Antioxidant capacity

Source		Sum of Squares	df (Degrees of freedom)	Mean Square	F Value	p-value Prob > F	
Model		1.39	5	0.28	25.55	<0.0001	Significant
A-Pressure		8.94E-3	1	8.94E-3	0.82	0.3878	
B-Temperature		0.34	1	0.34	31.39	0.0003	
C-Flow rate		0.18	1	0.18	16.22	0.0030	
AC		0.64	1	0.64	58.81	<0.0001	
BC		0.22		0.22	20.49	0.0014	
Residual		0.098	9	0.011			
	Lack of Fit	0.096	7	0.014	13.93	0.0686	Not significant
	Pure error	1.96E-3	2	9.82E-4			
Cor Total		1.48	14				

Table S3. ANOVA analysis for astaxanthin concentration

Source		Sum of Squares	df (Degrees of freedom)	Mean Square	F Value	p-value Prob > F	
Model		0.1343	4	0.03358	35.03	<0.0001	Significant
A-Pressure		0.0429	1	0.04289	44.75	<0.0001	

B-Temperature		0.0006	1	0.00063	0.66	0.4349	
C-Flow rate		0.0654	1	0.06538	68.21	<0.0001	
AC		0.0254	1	0.02541	26.51	0.0004	
Residual		0.0096	10	0.00096			
	Lack of Fit	0.0093	8	0.00116	8.68	0.1073	Not significant
	Pure error	0.0003	2	0.00013			
Cor Total		0.1439	14				
